# Peri-Implant Bone Behavior after Single Drill versus Multiple Sequence for Osteotomy Drill

**DOI:** 10.1155/2018/9756043

**Published:** 2018-04-11

**Authors:** Sergio Alexandre Gehrke, Raphaél Bettach, Jaime Sardá Aramburú Júnior, Juan Carlos Prados-Frutos, Massimo Del Fabbro, Jamil Awad Shibli

**Affiliations:** ^1^Department of Research, Biotecnos Research Center, Montevideo, Uruguay; ^2^New York University, New York, NY, USA; ^3^Private Practice, Gretz-Armainvilliers, France; ^4^Veterinary Program, Itapiranga Faculty, Itapiranga, SC, Brazil; ^5^Department of Medicine and Surgery (Stomatology Area), Rey Juan Carlos University, Madrid, Spain; ^6^Research Center in Oral Health, Department of Biomedical, Surgical and Dental Sciences, IRCCS Istituto Ortopedico Galeazzi, Università degli Studi di Milano, Milano, Italy; ^7^Department of Periodontology and Oral Implantology, University of Guarulhos, Guarulhos, SP, Brazil

## Abstract

**Objectives:**

The present study aims to compare the drilling protocol effect on osseointegration event in three commercially available titanium dental implants with different drill protocol using a rabbit tibia model.

**Materials and Methods:**

Three different drilling sequences were compared as follows: drilling sequence using a single unique drill of 4.2 mm conical implant (Group 1), drilling sequence using 3 consecutive cylindrical drills for a 4.1 mm cylindrical implant (Group 2), and drilling sequence using 3 consecutive conical drills for a 4.3 mm conical implant (Group 3). For each group, 18 drilling procedures and implant placements were performed, totalizing 54 commercially available titanium dental implants. The samples were removed 6 weeks after implantation. Resonance frequency analyses (RFA) were performed immediately after the implantation, and at 6 weeks removal torque test (RTt) and histological analysis were performed.

**Results:**

The RFA measured showed statistical difference between the groups in time 1 and no significant statistical differences in time 2 (*p* > 0.05). In the RTt no significant difference was found between the 3 groups tested. Histomorphometric analysis showed no significant difference between groups in the bone-to-implant contact% (*p* > 0.05).

**Conclusion:**

In the present preclinical study, osteotomy using a single bur did not show differences regarding the proposed and evaluated tests parameters for assessing the peri-implant behavior.

## 1. Introduction

The rehabilitation of tooth loss with dental titanium was documented and shown to have more than 98% of success rate [1, 2]. Osseointegration is the first step to the success of this type of treatment, which was defined as a direct contact between the bone tissue and the implant without the presence of fibers (soft tissue) [3]. It has been suggested that success of osseointegration is related to 6 main criteria: material biocompatibility, implant design, surface morphology, conditions of the implanted tissues, surgical technique, and loading conditions [4]. Among these, the excessive surgical trauma, prosthesis overload, misfit of suprastructures, or implanted area with infection can be considered the critical modifiable factors [5, 6].

Minimizing surgical trauma to bone tissue during the osteotomy is a controllable factor and may contribute to the osseointegration success [7]. Therefore, while drilling the bone, the temperature control during the osteotomies, due to attrition of burs, can cause tissue alterations and cells death, mainly damage in the organic portion of bone tissue [8, 9], may interfere directly in the process of bone healing (osseointegration), and can induce the crestal bone loss and to influence implant survival [10–13].

On the other hand, regarding the implanted material, there is still a lack of knowledge about the events related to a bone response in relation to the different surface types and which would be the most appropriate. Some steps of the actual biological events are the initial activation of bone healing around the implants, such as protein adsorption, interaction between cells and implant surface, migration and differentiation of progenitor cells, and tissue formation at the bone-implant interface, supposed to be affected by the surface morphology of the implant and its physicochemical structure [14–17]. In addition, the initial stability of the implant is directly linked to its macro design, such as cylindrical or tapered design, its length and diameter, and the type of turns and the distance between them. All these factors may act positively or negatively on implant locking in bone tissue [18]. In this regard, Gehrke et al. [19], published a study that showed measurements of the insertion torque value (ITV), implant stability quotient (ISQ), and precision of osteotomy using conventional and simplified (a single drilling step is used) drilling systems, and the evaluation showed that the hole quality and the ITV promote a significant increase in the primary stability of the implants. Therefore, the system using a single drill for the osteotomy showed significantly higher ITV and ISQ than the systems tested using a multiple-drill sequence for the osteotomy.

Recently an increasing interest has been shown in the scientific community regarding the investigation of different drills design and osteotomy protocols, its results on the bone trauma control, and consequently its effects on the bone healing [20–23]. However, there is little information available in the literature about what should be the ideal progression for increasing the diameter during osteotomy and/or whether it should be progressive. However, it was assumed that the osteotomy was performed by incremental steps, increasing the drilling diameter slowly and thus minimizing trauma to the bone tissue. There is little evidence in the literature about the effects of different milling protocols and which would be ideal and therefore less traumatic. However, Gehrke [22] showed in a histological study that the use of new drill (discardable drill) can promote better results in comparison with multiple use drills. More recently, Bettach et al. [24] published a human study where it became evident that the use of a drilling protocol with a single drill can present a high success rate of osseointegration of the implants [24]. This type of protocol can bring some important advantages from the point of view of required working time and manipulation over bone tissue (perforation) when compared to traditional staggered protocols, which require obviously longer time steps. Thus, there should be a balance between the precision in the positioning required by the implant in terms of the inclination, diameter, and shape of the osteotomy, thus seeking an ideal anchorage (stability) of the implants and rationing of the total time necessary to perform it. In addition, another proposed care was that the final drilling bur should be shorter; thus, hypothetically, it decreases the exposure for an extended time and, consequently, the possibility of generating more heating to the bone tissue. In this sense, the proposition to investigate the reduction of the number of drills during implant osteotomy, using a single drill with high cutting power, was analyzed and compared with the conventional sequences using multiple drills. This new methodology and technology tested aim to provide results similar to the conventional sequence (multiple) used until today [20–23].

The purpose of this study was to compare, through biomechanical and histological analysis, the effects on the osseointegration event using a reduced protocol for osteotomy (only one drill) with the conventional drilling protocol (multiple) for implant osteotomy, using a rabbit tibia model.

The null hypothesis was that the use of a reduced protocol for osteotomy (one drill) did not affect the osseointegration process of the implant when compared to the osseointegration of implants installed using conventional osteotomy (multiple drills).

## 2. Materials and Methods

**Figure 1:**
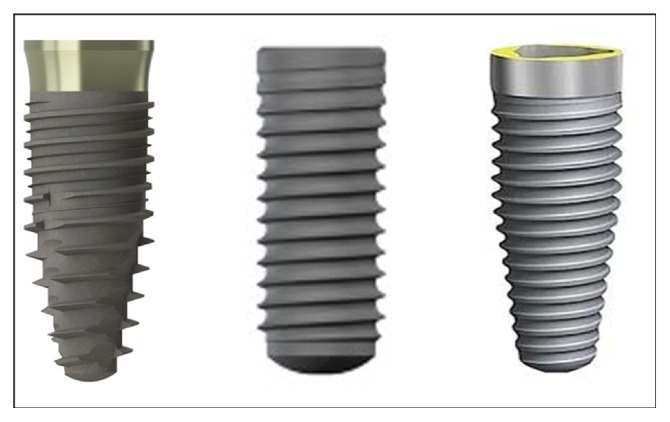
Images of the implants used in the study.

**Figure 2:**
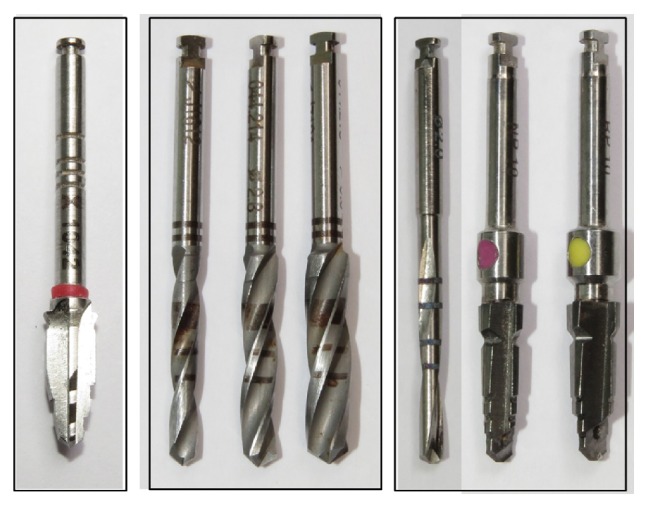
Drill sequences used for osteotomy in the groups 1–3, respectively.

Fifty-four commercially titanium dental implants with 3 different designs (Figure 1) and drill sequences for osteotomy protocol (Figure 2) were divided into 3 groups (*n* = 18 implants per group): 

*Group 1.* One drill for conical implant with Ø4.2 mm and 10 mm in length (Implants Diffusion International, Montreuil, France), with recommended speed in 1500 rpm.

*Group 2.* Sequential drills for cylindrical implant with Ø4.1 mm and 8 mm in length (BoneLevel, Straumann, Basel, Switzerland): Ø2.2 mm (800 rpm), Ø2.8 mm (600 rpm), and Ø3.5 mm (500 rpm) [25].

*Group 3.* Sequential drills for conical implant with Ø4.3 mm and 8 mm in length (NobelReplace® implant, Nobel Biocare, Göteborg, Sweden): Ø2 mm (2000 rpm), Ø3.5 mm (800 rpm), and Ø4.3 mm (800 rpm) [26].

In Group 1, the implant surface was prepared by sandblasting acid (SLA) using blasting with aluminum oxide plus acids attack and then a thermic treatment (IDI, Montreuil, France); in Group 2, the implant surface is treated by a SLA procedure with aluminum oxide for blasting plus acids attack (Straumann, Basel, Switzerland); and, in Group 3, the implants were treated by anodization method (TiUnite®, Nobel Biocare, Sweden). All implants were purchased from their local resellers in the same conditions under which they are marketed for clinical use.

### 2.1. Animals and Surgical Procedure

**Figure 3:**
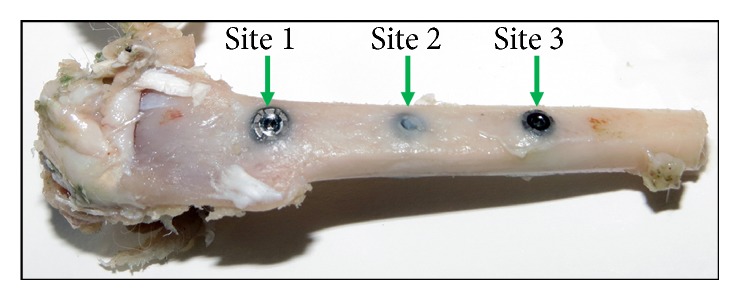
Image of the tibia showing the site numeration used to the implants distribution.

Nine New Zealand white adult rabbits weighing between 4 to 4.5 kg were used for the present preclinical study. This study was approved by the Ethics Committee of the Itapiranga Faculty, Itapiranga, Santa Catarina, Brazil (#004-09-2015). This type of animal presents adequate conditions for the evaluation of the healing of the bone tissue around implants [27, 28] and is frequently used for experimental preclinical studies [23]. To anesthetize the animals, ketamine 35 mg/kg intramuscular (Agener Pharmaceutical, Brazil) was used plus Rompun 5 mg/kg (Bayer, São Paulo, Brazil). Additionally, Acepran 0.75 mg/kg (Univet, São Paulo, Brazil) was used as tranquilizer. Besides, a local anesthetic (3% Prilocaine-felypressin, Astra, Mexico) was subcutaneously administered near of the location of implantation to make vasoconstriction and control the pain. Then, a tissue incision was made to access the bone, the flap was lifted exposing the bone tissue, and the perforations were performed under abundant irrigation using the milling sequence determined and previously written for each implant model (each group). One implant of each group was placed in each tibia (3 per tibia), with the sites being numbered from proximal to distal as 1–3 (Figure 3), and distributed equally for each group. The implants were anchored bicortically; however the cervical portion of all implants was positioned at the level of the cortical bone. The control of the torque at the level of 20 ± 5 Ncm during the implant insertion was performed by a manual torquimeter and, finally, the implant stability quotient was measured. The suture using a 5-0 nylon were performed with individual simple points. Postoperatively, an antibiotic dose was administrated (600,000 IU Benzetacil). In the postsurgery, all animals received the care standardized by the veterinary hospital, that is, individual places with 12-hour cycles of light/dark, 21°C of temperature, and ad libitum diet. The postoperative period of the animals was within normality, that is, without complications or adverse events. Six weeks after the surgery, all were euthanized using an intravenous overdose of the anaesthetics (2 ml of ketamine plus 1 ml of xylazine). The 2 tibias were removed and packed in bottles with 10% formalin solution. Then, they were taken to the laboratory (Biotecnos, Santa Maria, Brazil) for immediate analysis.

### 2.2. Resonance Frequency Analysis

**Figure 4:**
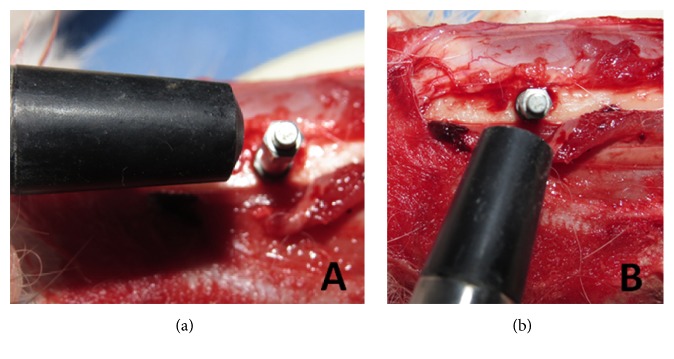
Images showing the 2 directions of the ISQ measurements of each implant. (a) Proximal to distal and (b) lateral to medial.

Resonance frequency analysis (RFA) was performed to evaluate the implant stability. A Smartpeg™ (Integration Diagnostics AB, Göteborg, Sweden) was screwed in the implants with approximately 5 N. The implant stability quotient (ISQ) was measured using the Osstell™ Mentor (Integration Diagnostics AB, Göteborg, Sweden), with the sensor being positioned at a distance of 2 or 3 mm from the Smartpeg. The RFA of each implant was measured immediately after the installation and after the sacrifice (6 weeks). The ISQ data used for each implant sample was an average of the collected value of 2 directions (proximal to distal and lateral to medial) (Figure 4).

### 2.3. Removal Torque Test

**Figure 5:**
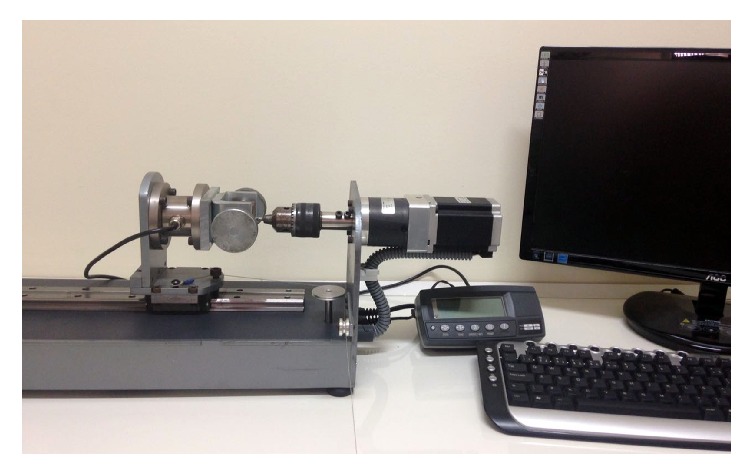
Image of the computerized torque machine used in the removal torque test.

Nine implants (3 per group) were removed in contra-torque. These implants were removed in site 1 (more proximal site) of the tibia by lot between the groups. A computerized torque testing machine (CME, Técnica Industrial Oswaldo Filizola, São Paulo, Brazil), used in other studies and developed by our group, was used in the present study [29] (Figure 5). The test speed used was 4 rpm, the maximum torque values, measured upon initiating the reverse rotation of each implant, were recorded, and the mean torque for each group was calculated.

### 2.4. Histomorphometric Analysis

Forty-five osseointegrated implants (15 per group) were treated by a dehydration process in sequential alcohols concentration (50 to 100%) and embedded historesin (Technovit 9100 VLC, Kulzer, Germany). Then, the blocks with the samples were cut into the portion corresponding to the center of each implant in slices of ~50 *μ*m thick using a micrometric cutter (Isomet 2000, Buehler, Germany). The histological slides were set, abraded, and polished by a sequence of sticks up to ~30 *μ*m thick and finally stained with picrosirius hematoxylin and analyzed histologically.

In each sample the bone tissue surrounding the implant in the cortical bone portion was histologically evaluated, and the percentage of bone-to-implant contact (BIC%) was made using a light microscope (EOS 200, Nikon, Tokyo, Japan). The measurements of BIC% were performed using the software Image Tool version 5.02 for* Microsoft Windows*™ on the digitalized images. BIC% was calculated as the percentage of bone that was in direct contact with the implant surface.

### 2.5. Data Analysis

The data were longitudinally compared among the groups with the Friedman test and one-way analysis of variance (ANOVA) test for repeated measures. The Mann–Whitney *U* test was used for the comparative analysis among the 3 groups in the same test. These statistical analyses were made with the computational program GraphPad Prism 5.01 (GraphPad Software Inc., San Diego, CA, USA). The level of significance was set at *α* = 0.05.

## 3. Results

In performed postoperative controls, no healing problem was observed, presenting adequate evolution in the weekly evaluations. After sacrifice (6 weeks), all implants were osseointegrated. Six weeks after the surgical implantation, all implants were osseointegrated.

### 3.1. Resonance Frequency Analysis (RFA)

**Figure 6:**
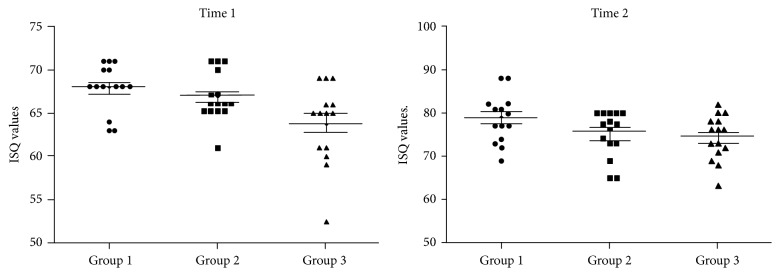
Graphs of the ISQ values distribution of the 3 groups in the 2 times (baseline and 6 weeks).

The measured values and statistical analysis of RFA for the 2 times measured of the 3 groups are shown in Table 1. Performing the statistical test within the times (baseline and 6 weeks) among the groups studied, the values showed statistically significant differences in time 1 (*p* = 0.005) and no difference in time 2 (*p* = 0.068). The data distribution in each time of the groups is presented in the graphs of Figure 6.

### 3.2. Removal Torque Test (RTt)

In RTt, all samples presented a good stability in the bone tissue. The mean resistance to removal torque values and standard deviation was 95.7 ± 3.21 N for Group 1, 91.0 ± 2.65 N for Group 2, and 91.0 ± 3.61 N for Group 3. The statistical test showed no differences between the groups (*p* = 0.622).

### 3.3. Histological Analysis

**Figure 7:**
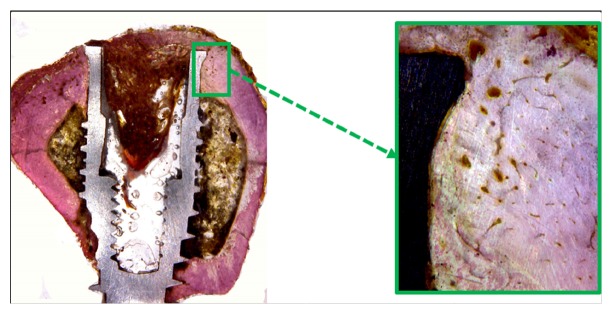
Histological pictures showing the bone healing around the implant after 6 weeks of Group 1. Magnification: 4 and 100x, respectively. Picrosirius-hematoxylin staining.

**Figure 8:**
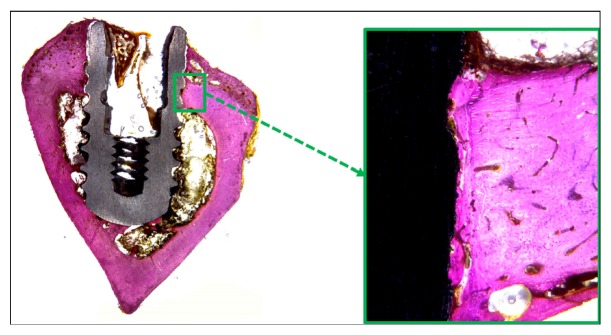
Histological pictures showing the bone healing around the implant after 6 weeks of Group 2. Magnification: 4 and 100x, respectively. Picrosirius-hematoxylin staining.

**Figure 9:**
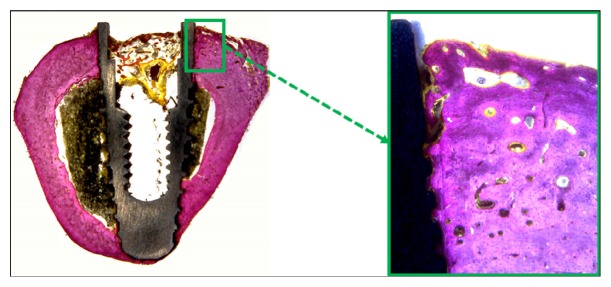
Histological pictures showing the bone healing around the implant after 6 weeks of Group 3. Magnification: 4 and 100x, respectively. Picrosirius-hematoxylin staining.

Histological observations showed adequate bone organization and mineralization around the implants at 6 weeks in all groups (Figures 7–9). The BIC% values measured in the cortical bone portion were 71.7 ± 2.94% for Group 1, 70.8 ± 2.43% for Group 2, and 70.8 ± 3.30% for Group 3. The data analysis did not show statistical differences between the 3 groups (*p* = 0.644).

## 4. Discussion

Recently, a clinical study evaluation of 350 implants installed in several clinical procedures showed excellent results using a single drill system for osteotomy, with 98% of implant survival [26]. Then, Gehrke et al. [30] investigated the possible relationship between this good result and the bone heat generation during the osteotomy (less surgical trauma) and concluded that this drilling system (using one drill), prepared to perform the osteotomy for implant placement in a single drilling maneuver, did not provide temperature rise in bone tissue compared to drilling systems using a stepped sequence (multiple drills) for osteotomy of the implant bed. In this sense, the present biomechanical and histological investigation in animals was developed to check and conclude if this protocol using a single drill for osteotomy did not change the osseointegration events and so this might be a possible explanation for the success of this technique.

In this histological study, we evaluated the bone response (osseointegration) of three different implant models with different osteotomy drilling systems. The findings showed that the use of only one drill for the osteotomy provided similar biomechanical and histological response than using a conventional multiple drilling. In a histological evaluation, several studies showed that bone tissue behavior in implants placed with a simplified protocol is similar to the conventional protocol using multiple sequential drills [20–23]. However, even if the considerations seem obvious, the differences between the osteotomy protocols should be considered as a possibility of reducing the surgical trauma during the installation of the implants.

Several investigations have studied the effects of macro design of implants with regard to the healing and stability events [31–33]. These modifications in implant macro design at the beginning were proposed to accelerate the osseointegration of the implants increasing the initial stability and later increase the rate of contact between bone and implant after the period of bone healing, which benefits the distribution of the loads generated when these structures enter into a masticatory function [34]. In the present study, three implant designs were used and, although they presented a great variation in their design, the response was quite similar between the groups. Thus, it would be possible to suggest that osteotomy may be the determining factor for obtaining one of the main requirements for the success of osseointegration, the initial stability.

By making a relation between the reaction provided by the type of osteotomy performed and the surgical trauma on the cortical bone tissue, several considerations can be highlighted in order to reduce the possible effects of physical stress. To reduce surgical trauma during the osteotomy procedure, several points should be considered: the structural design of the cutter, such as cutting blade, blade angles, edging, and dimensions; the speed of rotation, the force to be exerted (applied pressure), the amount of irrigation, the maximum torque applied, and the use of sequential diameter drills or a single step [35, 36]. On the other hand, the variations presented by the locations where the osteotomies will be performed, such as bone tissue volume and density, and consequently the time required for osteotomy execution are factors that may interfere with the trauma generated during surgery for the installation of implants.

Two biomechanical tests, removal torque test (RTt) and resonance frequency analysis (RFA), were used to evaluate the 3 different implant designs used in this study. The RTt serves as a parameter to determine the resistance of the connection between the bone tissue and the implant [37, 38], and the high values of resistance in the counter-torque for implant removal indicate that there is a high density of bone tissue and a strong connection between bone and implant [38]. However, the RFA possibility of measuring implant stability at any time during implant treatment, without adding load, can be considered a noninvasive method for this type of evaluation [39, 40].

Gehrke and da Silva Neto [41] demonstrated in a clinical human study that the ISQ values have a direct relationship with the bone density (maxilla and mandible), even though the measurements show stability values in short periods, and the authors concluded that the RFA method is quite useful as a tool for clinical and noninvasive research and may help to demonstrate the behavior of implants and their relationship with peri-implant tissues, especially with respect to bone tissue. In addition, the clinical observations indicated that the final healing time was different between the study participants and the local conditions evaluated. Concerning the evolution of bone healing (osseointegration), studies have shown that there is a growing increase in SSI measurements, which was ~300 Hz per week [42]. The measurements in present study showed statistical differences between the groups of ISQ values and baseline values (time 1). However, in time 2 (6 weeks), no differences were found. Based on an overall average of the ISQ measured among the first and second time, points of all groups, with this variation of RFA, increased by 14.8%.

The counter-torque analyses of the implants are invasive biomechanical tests which, as previously described, can demonstrate the strength of the union between the implant and the bone and do not show several differences of the values in the same animal model [29]. Because the test requires destruction of the study specimens, in the present study the measurements were performed in triplicate and the values showed no significant difference between the groups. On the other hand, high counter-torque indices were observed in all samples of the 3 groups studied, and the results found were consistent with the results of other published studies [29] and confirm the good surface quality of the implants used in this study. In the present study, a fully computer-controlled counter-torque machine has been used, and it has been used in other studies [29], thus eliminating the possibility of any distortion in the measurements caused by the operator.

Regarding the surfaces of the implants used in the present study, basically the surfaces of Groups 1 and 2 are very similar, being both SLA surface, whose excellent osseointegration of implants is evidenced by several studies and which offers predictable long-term results [43, 44]. Similarly, the surface presented by Group 3 implants (TiUnite) has a proven high success rate [45, 46]. Therefore, the results presented in the this study probably had the same stimulus intensity regarding surface treatment in the 3 proposed groups.

Although the results of this technique are similar to those found in conventional implants systems, further studies on the use of single drill for osteotomy should be performed to investigate the lifetime, the wear of this drills, and the influence on the implant osseointegration.

## 5. Conclusion

Within the limitations of this animal study, the findings showed that a single drill system did not change the biomechanical and/or biological of peri-implant tissue response more than a conventional drilling sequence does while preparing implant site and may be considered as safe as the latter. The measured values in all proposed tests showed similar results for the three implant designs tested in the present study.

## Figures and Tables

**Table 1:** Friedman test of ISQ analysis and measurements at baseline (initial) and at 6 weeks. Results as mean and medians. Mann–Whitney *U* test to compare intragroups (*p* < 0.05).

ISQ value	Baseline		6 weeks		*p* value (intragroup)
	Mean ± Sd	Median	Mean ± Sd	Median	
Group 1	67.9 ± 2.69	68	78.7 ± 5.41	79	<0.0001
Group 2	66.8 ± 2.83	66	75.1 ± 5.26	77	<0.0001
Group 3	63.8 ± 4.36	65	74.3 ± 5.18	76	<0.0001
*p* value (intergroup)	0.005		0.068		
